# The social factors behind the mask: contextual effects on trait impressions from faces wearing a face mask

**DOI:** 10.1186/s41235-024-00570-w

**Published:** 2024-06-27

**Authors:** Matilde Tumino, Luciana Carraro, Luigi Castelli

**Affiliations:** Department of Developmental and Social Psychology, University of Padova, Via Venezia 8, 35131 Padua, Italy

**Keywords:** Trait impressions, Contextual factors, Face masks, COVID-19, Social evaluation

## Abstract

The presence of face masks can significantly impact processes related to trait impressions from faces. In the present research, we focused on trait impressions from faces either wearing a mask or not by addressing how contextual factors may shape such inferences. In Study 1, we compared trait impressions from faces in a phase of the COVID-19 pandemic in which wearing masks was a normative behavior (T1) with those assessed one year later when wearing masks was far less common (T2). Results at T2 showed a reduced positivity in the trait impressions elicited by faces covered by a mask. In Study 2, it was found that trait impressions from faces were modulated by the background visual context in which the target face was embedded so that faces wearing a mask elicited more positive traits when superimposed on an indoor rather than outdoor visual context. Overall, the present studies indicate that wearing face masks may affect trait impressions from faces, but also that such impressions are highly flexible and can significantly fluctuate across time and space.

## Significance statement

During the COVID-19 pandemic, the use of face masks has affected our daily lives in several ways, including social perception processes. These two studies investigated the impact of face masks on trait impressions from faces, highlighting the critical role of contextual factors.

The first contextual factor addressed in the present research is related to changes in the normative context. In a quasi-experimental study, we found that the trait impressions triggered by faces wearing the mask became less positive in a time period in which mask-wearing was less common and rules relaxed (T2), as compared to those observed in a period in which mask-wearing was a widespread behavior also supported by law requirements (T1).

The second contextual factor that was examined is the visual context in which masked and unmasked targets were presented. In Study 2 we found that trait impressions from faces wearing the mask were more positive when targets were presented in indoor rather than outdoor visual contexts. Conversely, unmasked faces elicited more positive impressions in outdoor rather than indoor visual contexts.

Overall, this research contributes to our understanding about how trait impressions from faces wearing a protective mask can fluctuate over time and space, shedding light on the complexity of trait impressions from faces impressions in the midst of a global pandemic. These findings are not only relevant for understanding the impact of face masks but also for broader considerations on the influence of contextual factors on social perception.

## Introduction

Since the outbreak of the COVID-19 pandemic research has started to intensively study the impact of face masks on several socio-cognitive processes involved in the perception of individuals around us, including emotion recognition (Carbon, 2020; Rinck et al., 2022), categorization (Castelli et al., 2022), and identity recognition (Carlaw et al., 2022; Carragher & Hancock, 2020; Wong & Estudillo, 2022). Another widely addressed issue has been related to the possible effects of wearing a face mask on trait impressions. Upon seeing a face, people rapidly make inferences about the personality characteristics of the person portrayed (Todorov et al., 2015), and these inferences, in turn, are associated with relevant behavioral consequences (e.g., Todorov et al., 2005). The research about trait impressions triggered by faces either wearing face masks or not has typically assessed evaluations along the key dimensions that characterize prevalent models of social perception (Brambilla et al., 2021a, 2021b; Fiske et al., 2007; see also Oosterhof & Todorov, 2008), namely competence, sociability, and morality. Whereas competence mainly refers to the possession of intellectual and practical skills, sociability and morality (e.g., trustworthiness, altruism) are more related to the functioning in social relationships. Critically, the results reported in the literature about the impact of face masks on trait impressions from faces appear to be often inconsistent across studies. Several studies found an increased positive perception of faces wearing a face mask as compared to faces without the mask (Castelli et al., 2022; Di Crosta et al., 2023; Guo et al., 2022; Oldmeadow & Koch, 2021; Olivera-La Rosa et al., 2020). Other studies, however, reported no significant effect, mixed findings, or even an opposite pattern (i.e., more positive ratings for faces without the mask; Bennetts et al., 2022; Biermann et al., 2021; Grundmann et al., 2021; Takehara et al., 2023; Twele et al., 2022). This makes it important to highlight some overarching factors that might shape the strength, and, possibly, the direction of the effects.

Overall, impression formation from faces can be driven by both perceptual cues and conceptual information (Sutherland & Young, 2022). Accordingly, two different theoretical approaches can be taken in order to frame the effects of face masks on trait impressions from faces. On the one hand, face masks inevitably hinder relevant parts of the face and therefore the perceptual information available for making any inference is reduced (i.e., the area around the mouth). As a consequence, the occlusion of the lower part of the face may potentially alter the inferential processes that typically occur in the case of uncovered faces (see Ganel & Goodale, 2022; Oliveira & Garcia-Marques, 2022). In the present work, we will not focus on these relevant processes, but rather on the role of more conceptual information. Indeed, during the various waves of the pandemic, when people formed impressions from masked faces it is likely that they could hardly disregard the deepest meaning of wearing masks, both in terms of personal protection from the virus and communal behavior aimed at safeguarding the health of others (see Olivera-La Rosa et al., 2020). In particular, in most countries, since the onset of the pandemic, injunctive norms prescribed the use of face masks, and strict law requirements were in place. At the same time, descriptive norms (i.e., norms based on the assessment of how people actually behave; Cialdini et al., 1990) signaled that wearing face masks was the typical, widespread, and appropriate behavior. Hence, mask wearers could be appraised as individuals complying with the prevalent norms and this, in turn, could affect the trait inferences drawn from their faces. According to this rationale, changes in the normative context could thus lead to shifts in the personality characteristics that are inferred from the face. This hypothesis has been addressed in the present work in which, in a quasi-experimental design, we compared the trait impressions provided by the same respondents about the same face stimuli—either wearing or not face masks – at two different points in time. In particular, we relied on judgments assessed during an intense phase of the pandemic in Italy (i.e., December 2021) when both injunctive and descriptive norms supported the use of face masks (see Castelli et al., 2022) with judgments provided roughly one year later (i.e., December 2022) when governmental regulations softened, and the large majority of people no longer wore face masks. We expected that these contextual changes would be significantly associated with a reduction in the positivity of the trait impressions from faces wearing masks. Specifically, more positive trait impressions from masked (vs. unmasked) faces were predicted at T1 than at T2. To the best of our knowledge, only two studies in this research area have included temporal comparisons. Takehara et al. (2023), in a study conducted in Japan, found that higher trustworthiness ratings were provided toward faces without the mask and this pattern was stable across time (i.e., September 2020 vs April 2022). Bennetts et al. (2022) tested British participants in 3 different periods (i.e., between June 2020 and August 2021) and reported no effect of masks on judgments about trust and competence, as well as no effect of time of assessment. Overall, these studies provide no evidence about possible temporal fluctuations in the trait impressions from faces wearing protective masks. Most importantly, these studies assessed the trait impressions from faces provided by different samples in each wave of data collection, thus preventing a precise evaluation of eventual temporal shifts. Study 1 directly addressed this issue by testing the same sample of respondents at two distinct points in time that were characterized by a different normative context.

### Trait impressions from faces as a function of the proximal context

Contextual influences can be framed at different levels of specificity. In relation to mask-wearing, as discussed above, they can be conceived in terms of the broader conditions affecting the life of the individuals (e.g., the infection rate or the presence of law requirements). However, contextual influences can also operate at a more proximal level, namely as a function of the specific visual context in which we encounter people. Past research, for instance, has shown that the activation of stereotypical knowledge is dependent upon the context background in which members of stigmatized outgroups are presented (Barden et al., 2004; Wittenbrink et al., 2001; see also Freeman et al., 2013). In a similar vein, the perception of facial emotion appears to be shaped by the content of the surrounding scene (Righart & de Gelder, 2006, 2008). This implies that the very same stimulus can be differently appraised in different visual contexts. More relevant to the present work, recent research has nicely demonstrated that also the evaluation of facial trustworthiness is significantly affected by the background visual context in which faces are presented (Brambilla et al., 2018; Mattavelli et al., 2022, 2023; see also Jenkins et al., 2011). In a typical experimental procedure, participants are presented with faces superimposed on different background visual contexts (e.g., neutral or threatening scenes) and required to categorize the faces as a function of their perceived trustworthiness (Brambilla et al., 2018). Notably, although no guidance was given about how to process the background visual context, faces appeared to be judged as less trustworthy when shown in threatening contexts, thus indicating that trait impressions from faces may incorporate information provided by the nature of the surrounding environment (Brambilla et al., 2018). Overall, this points to the importance of studying trait impressions from faces considering the surrounding visual context, and this approach also enhances the ecological validity of the findings given that we rarely encounter other individuals in a social vacuum (Hehman et al., 2019). Accordingly, it might be expected that the trait impressions from faces of individuals either wearing or not a face mask is sensitive to the visual context in which the faces are embedded. The adoption of protective measures is indeed differentially relevant as a function of the specific contexts. During a pandemic, whereas in closed spaces (e.g., in a shop) wearing a mask is maximally valuable in order to prevent the transmission of the virus from one person to another, in open and uncrowded contexts (e.g., in a park) it might be less so. Hence, in Study 2, which was carried out when strict norms about the use of face masks were still in place, we explored the impact of this additional factor in shaping trait impressions from faces. Participants were presented with masked and unmasked faces and the background visual context, that could either be the aisle of a supermarket or a wooded park, was manipulated. We expected to observe a stronger difference between the trait impressions from masked versus unmasked faces when they were presented in an indoor visual context, as compared to when faces were embedded in an outdoor visual context.

## Study 1

### Participants

We aimed to recruit as many of the participants who had taken part in a former study carried out in December 2021 (Study 1 reported in Castelli et al., 2022, T1). Data collection was carried out online through the Prolific platform (both at T1 and T2). Of the original 200 respondents in T1 (96 females, 101 males, 3 non-binary; *M*_age_ = 27.04 years, *SD*_age_ = 8.20, ranging from 18 to 63 years), 24 of them were no longer active on Prolific at the time of the present data collection which took place in December 2022 (T2).^1^ We invited all the remaining 176 original respondents and 169 completed the questionnaire (84 females, 81 males, 4 non-binary; *M*_age_ = 28.29 years, *SD*_age_ = 8.29, range 19–61 years; 84.5% of the original sample at T1). All participants were Italian native speakers and they all provided informed consent. The study was approved by the Psychology Ethics Committee at the University of Padova and it was carried out according to relevant guidelines and regulations.^2^

### Procedure

At T1 (Castelli et al., 2022) participants initially completed a memory confusion task (Taylor et al., 1978). The task first required participants to go through a presentation phase in which they were shown a sequence of twenty-four neutral sentences together with the picture of the face of the speaker who had allegedly pronounced each of them. Pictures of 8 different White male speakers were employed. Faces were retrieved from the Chicago Face Database (Ma et al., 2015) and on half of them a surgical face mask was digitally added using Adobe Photoshop. Afterward, participants were tested on their capacity to correctly match each sentence with the identity of the speaker. Results, that are not directly relevant to the aims of the present work, showed that participants spontaneously encoded the information about whether the speaker wore the face mask or not (for more details see Castelli et al., 2022). Personal attitudes toward the use of face masks were then assessed. Most relevant here, participants were finally asked to report the trait impressions in relation to each of the eight faces. Trait impressions were assessed along 5 dimensions: trustworthiness, morality, sociability, competence, and altruism. Responses were provided along a continuum ranging from 0 (= not at all) to 100 (= very much).

At T2 the memory confusion task was not administered and the attitudes toward the use of face masks were not assessed. Participants were only required to report trait impressions from faces. The same eight faces seen at T1, 4 wearing the face mask and 4 without the face mask, were presented. Pictures were displayed in a random order and, for each face, participants were required to report the perceived trustworthiness, morality, sociability, competence, and altruism. Responses, as at T1, were provided along a continuum ranging from 0 (= not at all) to 100 (= very much). Hence, the assessment of trait impressions from faces was identical at T1 and at T2. Finally, participants were asked to report demographics.

### Results^3^

Because the reported trait impressions from faces elicited by each target along the various traits were highly intercorrelated at both T1 and T2 (αs > 0.90), we first calculated 4 summary scores (i.e., trait impressions from faces with either the mask or not, separately at T1 and T2). These 4 scores were then analyzed through a 2 (Face mask: Present vs. absent) × 2 (Time: T1 vs T2) ANOVA with both factors varying within participants A main effect of the face mask emerged, *F*(1,166) = 104.0, *p* < 0.001, η^2^_p_ = 0.385, indicating that faces with a face mask elicited more positive trait inferences (*M* = 56.70, *SE* = 0.81) as compared to faces without the mask (*M* = 48.40, *SE* = 0.72). Also, the main effect of Time emerged, F(1,166) = 19.30,* p* < 0.001, η^2^_p_ = 0.104, indicating more positive impressions at T1 (*M* = 54.06, *SE* = 0.76) than T2 (*M* = 50.50, *SE* = 0.83). Most important for the aims of the present work, the main effect of Time was qualified by a significant interaction between Time and Face Mask, F(1,166) = 34.00, *p* < 0.001, η^2^_p_ = 0.170, indicating that the evaluation of targets wearing a face mask became less positive at T2 as compared to T1, *p*_*Bonferroni*_ < 0.001, upper and lower limits: 95% CI [5.03, 9.40], while the impressions of targets who did not wear the face mask remained far more stable with time, *p*_*Bonferroni*_, = 0.627, upper and lower limits: 95% CI [-1.53, 2.53]. We also carried mean comparisons at each time point. As for T1, masked faces were evaluated more positively (*M* = 60.14, SE = 0.91) as compared to unmasked faces (*M* = 48.22, *SE* = 0.90, *p*_*Bonferroni*_ < 0.001, upper and lower limits: 95% CI [9.74, 14.11]). A similar pattern emerged at T2. Indeed, masked faces were still evaluated more positively (*M* = 52.93, SE = 1.01) as compared to unmasked faces (*M* = 47.72, SE = 0.80, *p*_*Bonferroni*_ < 0.001, upper and lower limits: 95% CI [3.44, 6.99]).

In an exploratory way, we also analyzed the data considering the responses to the 5 traits separately. A 2 (Face mask: present vs. absent) × 5 (Trait: trustworthy, moral, sociable, competent, and altruistic) × 2 (Time of assessment: T1 vs. T2) ANOVA with all factors manipulated within participants was carried out. The results confirmed the abovementioned pattern of results which was not qualified by the three-way interaction involving the Trait factor, *p* = 0.512. Therefore, no other analyses were carried out on responses to each single trait.

## Discussion

Findings confirmed a change in the trait impressions from faces of individuals wearing a face mask reported during a period when the COVID-19 pandemic posed a significant threat, compared to a period when the impact of the pandemic on social life was noticeably reduced. Whereas at T1 wearing face masks was a common behavior that was enforced to limit the spread of the virus, such behavior was largely unusual at T2. This was reflected in a shift in the valence of the traits that were inferred from faces wearing the mask. In contrast, no relevant modification in the traits inferred from faces without the mask was detected. Although we had no specific hypothesis about eventual changes in the perception of faces without the mask, we may speculate that being confronted with unmasked faces represents the usual and common experience people are most familiar with. Even during the most dramatic phases pandemic, people were continuously exposed to unmasked faces within the family, during online meetings, or simply watching the television. Hence, traits inferred from faces without masks can be expected here to be more stable and less influenced by situational factors.

Overall, results are consistent with the idea that contextual factors may indeed shape trait impressions from faces, and fluctuations can be observed in response to cues that signal behaviors (i.e., wearing the face mask) that are appraised as either normative or not (or, at least, less normative) in a given temporal period. In Study 2, we addressed the role of contextual factors from another perspective, namely by varying the background on which face stimuli were presented. Because the study was carried out in a period in which wearing face masks was still mandatory in closed public spaces, we expected more positive trait impressions from faces with a face mask (vs. without) when they were presented superimposed on an indoor visual context as compared to an outdoor visual context.

## Study 2

### Participants

**Fig. 1:**
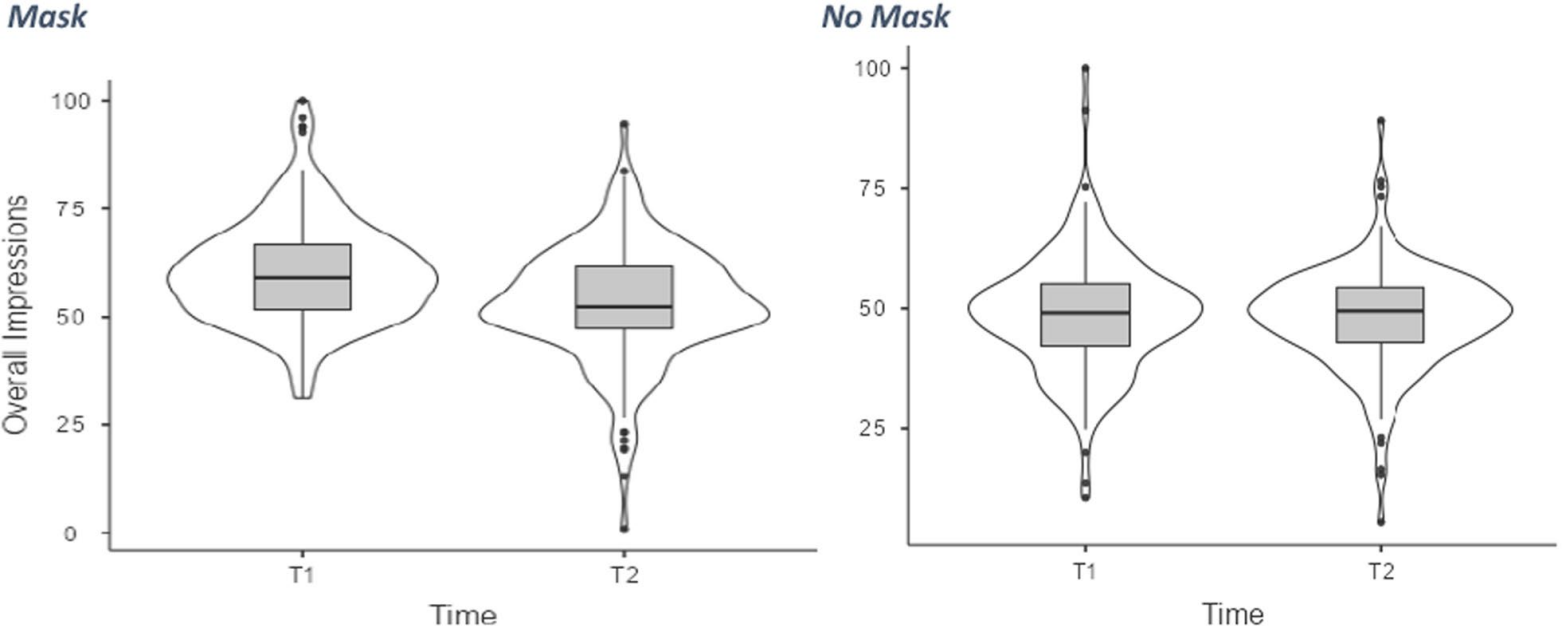
Two-way interaction between Time and Face Masks. Scores of our DV (Overall Impressions for masked and unmasked targets) range from 0 (strongly negative) to 100 (strongly positive)

We aimed to recruit as many participants as possible. Data collection was interrupted when norms related to the use of face masks changed, and wearing face masks became no longer mandatory in most closed public spaces. At that point, valid responses from 128 participants (89 females, 39 males, *M*_age_ = 29.93 years, *SD*_age_ = 11.80, range 18–69 years) were available. All participants were Italian native speakers. The study was conducted online on Qualtrics and started in February 2022. Participants were recruited through social networks, and they took part in the study voluntarily. All participants provided informed consent. The study was approved by the Psychology Ethics Committee at the University of Padova and carried out according to relevant guidelines and regulations. The sensitivity analysis showed that a sample size of 128, with effect sizes of ds > 0.25, could detect an effect with the probability of 80%-90% (and a type I error rate = 0.05). Hence, we can consider our sample size robust enough to inquire our hypothesis (Fig. 1).

### Procedure

The pictures of the faces of 12 male and female young adults were employed (retrieved from the Chicago Face Database, Ma et al., 2015). Only the face area was visible. The face images have been initially tested with a sample of respondents who did not take part in Study 2 (N = 30; 13 females, 14 males, 3 non-binary; *M*_age_ = 27.70 years, *SD*_age_ = 8.69). Respondents were shown all the pictures with neither the facemask nor any background visual context and asked to evaluate each of them along a series of dimensions: Competence, trustworthiness, sociability, altruism, morality, attractiveness, willingness to interact (i.e., all the trait dimensions that were assessed in the main study). Judgments were provided on a slider from 0 (“not at all”) to 100 (“totally”). A summary evaluation index was then computed for each face as the mean of the responses to all the trait judgments. On the basis of such summary evaluation indexes, it was possible to divide the face images into four subsets, so that the overall evaluation would be equivalent for both faces that would later appear in the main study with or without the mask, and with the indoor or outdoor background context. In particular, a 2 (Face mask in Study 2: present vs. absent) × 2 (Background in the main study: indoor vs. outdoor) Anova indicated that the interaction effect was not significant, *F* (1, 29) = 0.491, *p* = 0.498, ensuring that the expected effect in the main study could not be attributed to a priori differences in the stimulus materials.

**Fig. 2:**
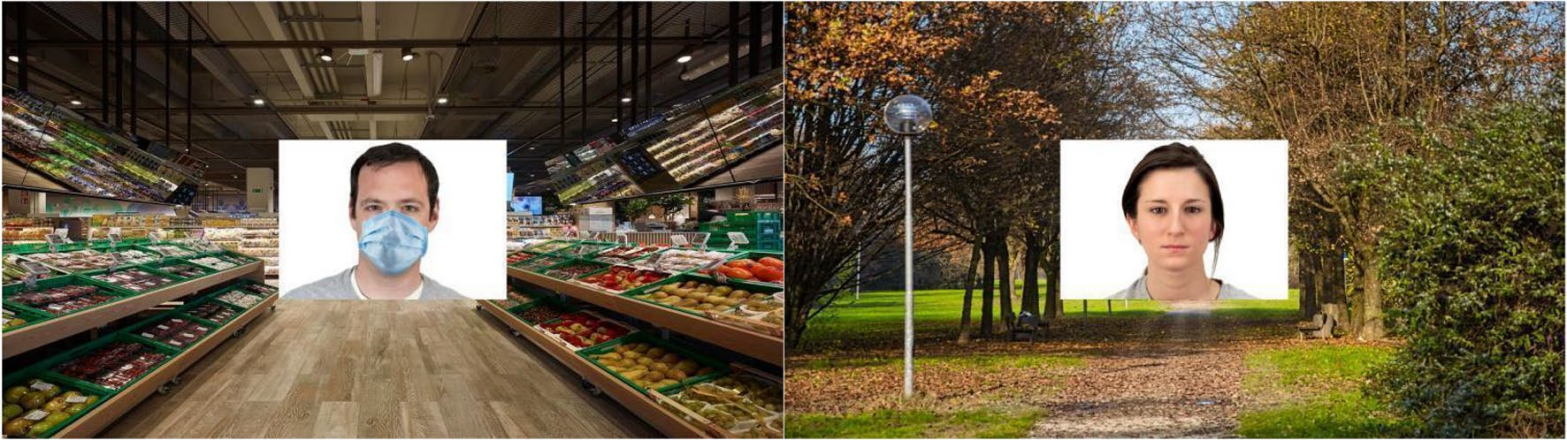
An example of a masked face presented in an indoor visual context (supermarket) and an example of an unmasked face presented in an outdoor visual context (park)

Face masks and background visual context were digitally added to the face stimuli used in Study 2. More specifically, half of the targets were wearing a face mask whereas the other half had no face mask superimposed. In addition, the background visual context was orthogonally manipulated (see Fig. 2): Half of the faces appeared in an indoor visual context (i.e., the aisle of a supermarket) and half in an outdoor visual context (i.e., a wooded park). Such background visual contexts have been selected as prototypical everyday life places in which the use of face masks was either mandatory (i.e. the supermarket) or not (i.e., the park).

For each target face, participants were invited to report on a slider from 0 (“not at all”) to 100 (“totally”) the trait impressions that were elicited when seeing the target face along 7 traits (competence, trustworthiness, sociability, altruism, morality, attractiveness, and willingness to interact), without any instruction about how to process the background visual context (see also Brambilla et al., 2018 for a similar procedure). Hence, we employed the same 5 traits included in Study 1 and 2 additional traits. Attractiveness and willingness to interact were included as further traits employed in relevant previous work about trait impressions from faces (e.g., Sutherland et al., 2013), and more specifically in several previous studies about the impact of face masks on impression formation (e.g., Diekhof et al., 2024; Hies & Lewis, 2022; Kühne et al., 2022; Oldmeadow & Koch, 2021). The presentation order of the faces was randomized.^4^

## Results

**Fig. 3:**
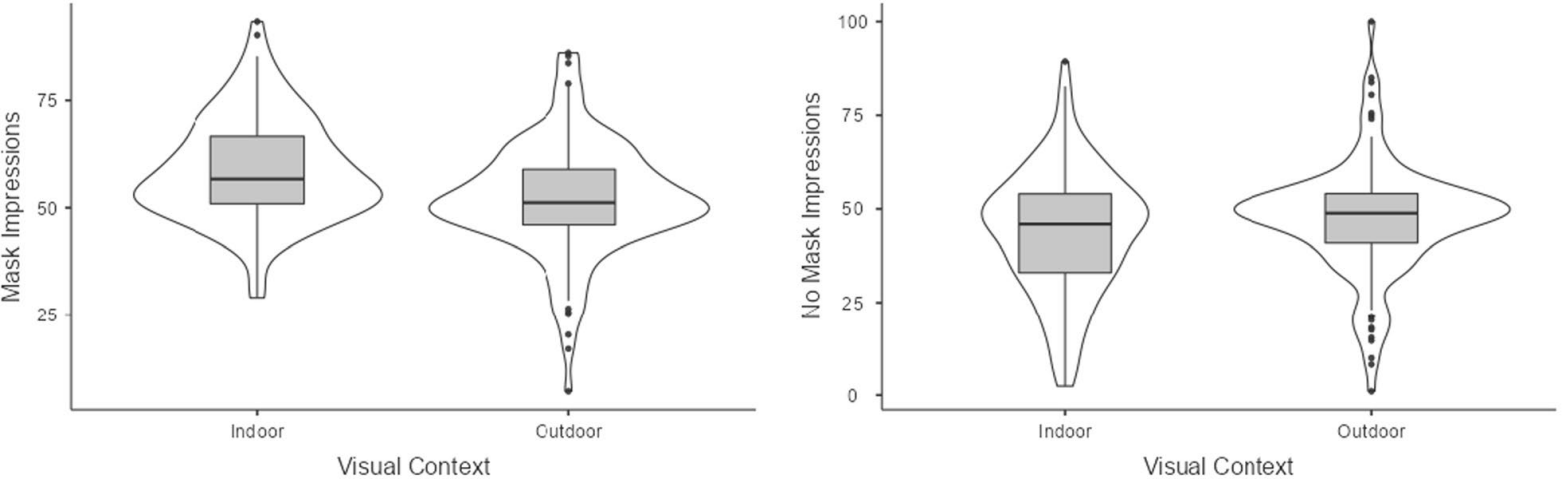
Overall impressions from masked and unmasked faces in indoor vs. outdoor visual context. Scores of our DV (Overall Impressions for masked and unmasked targets) range from 0 (strongly negative) to 100 (strongly positive)

Because the reported impressions elicited by each face along the various trait dimensions were highly intercorrelated (αs > 0.93), we first calculated summary scores. Four means were computed as a function of both the presence or absence of a face mask and the type of background visual context (i.e., with a face mask in an indoor visual context, with a face mask in an outdoor visual context, without a face mask in an indoor visual context, and without a face mask in an outdoor visual context). These scores were then analyzed through a 2 (Face mask: present vs. absent) × 2 (Visual context: outdoor vs indoor) ANOVA with both factors varying within-participants. A main effect of the face mask emerged, F(1,127) = 70.54, *p* < 0.001, η^2^_p_ = 0.357, indicating that faces with the face mask elicited more positive trait impressions as compared to the faces without the mask (see Table 1). The main effect of the visual context was also significant, *F*(1,127) = 5.78, *p* = 0.018, η^2^_p_ = 0.044, suggesting more positive trait impressions in the indoor visual context rather than the outdoor visual context. Most importantly, the interaction effect was also significant, *F*(1,127) = 60.48, *p* < 0.001, η^2^_p_ = 0.323. Post-hoc comparisons using the Bonferroni correction indicated the presence of more positive trait impressions in the case of masked faces presented in an indoor visual context (*M* = 58.79, *SE* = 1.06) than in an outdoor visual context (*M* = 51.96, *SE* = 1.13), *p*_*Bonferroni*_ < 0.001. In contrast, in the case of unmasked faces, trait impressions were more negative when faces were presented in an indoor visual context (*M* = 43.80, *SE* = 1.45) than outdoor visual context (*M* = 47.25, *SE* = 1.32), *p*_*Bonferroni*_ < 0.001 (see Fig. 3).

**Table 1:** Means (M), standard errors (SE) from the pairwise comparison with the Bonferroni correction

	MASK		NO MASK		INDOOR		OUTDOOR	
	Indoor	Outdoor	Indoor	Outdoor	Mask	No Mask	Mask	No Mask
Overall impressions	58.79 (1.06)	51.96* (1.13)	43.80 (1.45)	47.25* (1.32)	58.79 (1.06)	43.80* (1.45)	51.96 (1.13)	47.25* (1.32)
Trustworthy	61.76 (1.26)	55.76* (1.41)	44.12 (1.71)	48.24 (1.43)	61.76 (1.26)	44.12* (1.71)	55.76 (1.41)	48.24* (1.43)
Moral	57.36 (1.27)	55.53 (1.36)	43.53 (1.59)	47.87* (1.36)	57.36 (1.27)	43.53* (1.59)	55.53 (1.36)	47.87* (1.36)
Sociable	55.90 (1.12)	48.38* (1.23)	44.87 (1.35)	45.72 (1.36)	55.90 (1.12)	44.87* (1.35)	48.38 (1.23)	45.72 (1.36)
Competent	58.12 (1.18)	53.19* (1.26)	45.38 (1.59)	49.08 (1.40)	58.12 (1.18)	45.38* (1.59)	53.19 (1.26)	49.08 (1.40)
Altruistic	58.14 (1.31)	54.26* (1.33)	41.26 (1.77)	45.62 (1.49)	58.14 (1.31)	41.26* (1.77)	54.26 (1.33)	45.62 (1.49)
Attractive	57.84 (1.28)	43.26* (1.32)	41.69 (1.37)	44.43 (1.39)	57.84 (1.28)	41.69* (1.37)	43.26 (1.32)	44.43 (1.39)
Willingness to interact	62.42 (1.49)	53.34* (1.51)	45.78 (2.05)	49.84 (1.73)	62.42 (1.49)	45.78* (2.05)	53.34 (1.51)	49.84 (1.73)

*Significant *p*-value with the Bonferroni correction (alpha 0.05/28 comparisons)

For the sake of completeness, analyses were also carried out considering the 7 traits separately. To this end, a 2 (Face mask: present vs. absent) × 7 (Trait: competence, trustworthiness, sociability, altruism, morality, attractiveness, and approachability) × 2 (Visual context: outdoor vs indoor) ANOVA with all factors manipulated within participants was carried out. The main effect of Face mask, *F*(1, 127) = 70.54, *p* < 0.001, η^2^_p_ = 0.357, the main effect of the Visual context, *F*(1, 127) = 5.78, *p* = 0.018, *η*^*2*^_*p*_ = 0.044, and the interaction between Visual context and Face mask, *F*(1, 127) = 60.48, *p* < 0.001, *η*^*2*^_*p*_ = 0.323, were all significant, confirming the results emerged on the aggregated data presented above. The Trait factor yielded a significant main effect, *F*(6, 762) = 18.12, *p* < 0.001, *η*^*2*^_*p*_ = 0.125 and was also involved in 2-way interactions with the Visual context, *F*(6, 762) = 20.12, *p* < 0.001, *η*^*2*^_*p*_ = 0.137, and the Face mask factors *F*(6, 762) = 9.30, *p* < 0.001, *η*^*2*^_*p*_ = 0.068. These effects were qualified by a significant three-way interaction between Face mask, Visual context, and Trait, *F*(6, 762) = 11.30, *p* < 0.001, η^2^_p_ = 0.082. This suggests that the impact of the background visual context on trait impressions from faces with or without the mask was different as a function of the specific trait that was assessed. In order to better understand this complex interaction, pairwise comparisons were carried out, comparing the trait impressions from faces wearing a face mask in the indoor and outdoor visual context, as well as the trait impressions from faces without a face mask in the two visual contexts. After applying a Bonferroni correction, results showed more positive evaluations toward mask wearers in the indoor rather than outdoor visual context for all the dimensions, with the only exception of morality. In contrast, unmasked targets were significantly associated with only more positive evaluations along the morality dimension in the outdoor as compared to the indoor context, although the pattern (i.e., more positive evaluations in the outdoor context) was consistent across dimensions (see Table 1). In order to provide a comprehensive view of the data, we also compared masked and unmasked targets in the same visual context (Mask and No Mask Indoor, Mask and No Mask Outdoor, see Table 1). Results showed that in the indoor visual context, masked faces were always associated with more positive trait impressions as compared to unmasked faces. In the outdoor visual context, findings were more mixed, likely reflecting the fact that in open spaces wearing or not the face mask is somehow considered as less relevant.

## Discussion

The aim of Study 2 was to test the effect of the background visual context in which faces are presented. Findings showed that trait impressions from masked and unmasked faces changed as a function of the nature of the background visual context. In line with the hypotheses, masked faces triggered more positive impressions when embedded in an indoor visual context, such as the aisle of a supermarket, rather than in an outdoor visual context. The opposite pattern emerged in the case of unmasked faces. Overall, these results confirmed the effects of contextual information on trait impressions from faces (Brambilla et al., 2018; Mattavelli et al., 2022, 2023). Findings are consistent with the notion that trait impressions from faces are flexible and face-context integration processes may shape the final outcome.

### General discussion

Since the early stages of the COVID-19 pandemic, research started to focus on how covering the face with protective masks might affect trait impressions from faces. Notably, rather inconsistent findings have been reported so far. Whereas several studies reported bolstered positive impressions (e.g., Castelli et al., 2022; Di Crosta et al., 2023; Guo et al., 2022; Oldmeadow & Koch, 2021; Olivera-La Rosa et al., 2020), other studies did not observe a similar pattern (e.g., Bennetts et al., 2022; Biermann et al., 2021; Grundmann et al., 2021; Takehara et al., 2023; Twele et al., 2022). Cultural and individual difference factors can to a large extent account for this variability (see Leder et al., 2022; Swain et al., 2022). In the present research, we primarily aimed to explore how the variability in trait impressions from faces elicited by masked and unmasked faces might be, at least partially, affected by contextual factors.

Overall, results from both studies aligned with the evidence provided by previous studies (see Oldmeadow & Koch, 2021; Olivera-La Rosa et al., 2020) indicating that faces wearing a face mask were generally associated with more positive trait impressions as compared to faces without a face mask. Critically, however, this effect was neither stable in time nor impervious to situational cues, suggesting that the analysis of the impact of face masks on trait impressions would strongly benefit from a situated approach. As for the temporal dimension, trait impressions from faces wearing the mask changed across different phases of the COVID-19 pandemic. Indeed, adopting a within-participants design, Study 1 revealed a significant shift in trait impressions from faces wearing face masks as assessed at two different time points. While at T1 wearing face masks was a widespread behavior and it also signaled compliance with law requirements (i.e., it was a normative behavior), at T2 wearing face masks was far less common, less strict rules were in place. This likely accounted for the significant decrease in the positivity of trait impressions associated with mask wearers, consistent with the idea that the appraisal of social norms may affect how people react to mask-wearing (see Carbon, 2021). Importantly, no relevant changes were observed in the trait impressions associated with faces without masks. Overall, these results support the notion that contextual factors may play a crucial role in shaping trait impressions from faces so that the evaluation of people wearing face masks can fluctuate over time as a function of the normativity of the behavior. It could be reasonably expected that impressions about faces covered by a protective mask would be now increasingly less positive given that wearing face masks is currently a rare and not-prescriptive behavior. This is an empirical question that could provide additional insights about the intrinsically situated nature of trait impressions from faces.

The situated nature of trait impressions from faces, however, can also be considered at a different level of specificity. In Study 2, we focused on the effects of proximal contexts, namely the specific environment in which the face is embedded (see Brambilla et al., 2018; Mattavelli et al.,^2022^, ^2023^). Our overall results suggest that, in a time period in which the pandemic was still widespread, an indoor visual context (vs an outdoor visual context) intensified the positive trait impressions from faces wearing the mask, possibly due to the perception of increased safety and conformity to social norms. Indeed, even if participants were only invited to focus on the impressions elicited by each face, they spontaneously integrated the information provided by the background visual context in their judgments. A rich stream of research has already demonstrated that the presence of “accessories” such as facial hair, glasses, or jewels can affect trait impressions from faces (Sutherland et al., 2013; Vernon et al., 2014). Face masks seem to be no exception. Critically, however, findings from Study 2 indicate that the influence of such “accessories” is not necessarily fixed and predetermined, but it can be better understood by considering also the broader social context in which the face is embedded (see Hehman et al., 2019; Sutherland & Young, 2022). Future research will have to further address the specific role of visual context as a relevant source of variability in trait impressions from faces, exploring how face-context integration might also occur in a cross-modal fashion (e.g., when sounds evoke open environments vs indoor settings, such as singing birds vs the noise of a copier; see Brambilla et al., 2021a, 2021b). At a more general level, the observed effects could be expected to be larger when the face-context integration is facilitated. In Study 2, faces were simply superimposed to either the outdoor or indoor visual context in order to test whether the presence of a background was sufficient to alter trait impression from faces. However, more realistic stimuli in which the target is indeed *within* the scene should lead to a stronger face-context integration and, as a consequence, to potentially more impactful modulations.

As briefly mentioned in the introduction section, traits impression from faces are influenced by both perceptual cues (e.g., the size of the eyes) and more conceptual information, such as the context-driven social meaning attributed to specific features of the target (Sutherland & Young, 2022). We here primarily focused on how face masks impacted traits impressions from faces in varying contexts, but the outcome of this process may be hypothesized to exert an influence on the very same perception of the facial cues. As argued by Hassin and Trope (2000), we do not only “read from faces” (i.e., make attributions about personality traits), but we also “read into faces”, namely we modify the perception of the facial features in order to make it consistent with our overall impression about the target. Hence, the two processes are likely to be strictly intertwined and context-face integration may also involve what we actually perceive and remember about the facial cues of the target. Following the suggestions put forward by Brambilla et al. (2024), future studies should thus specifically address how the impact of contextual information could go well beyond impression formation processes. In this regard, data-driven approaches, such as the reverse correlation paradigm (Dotsch & Todorov, 2012), are good candidates for providing novel relevant insights.

Although the present studies provided consistent evidence about context effects on trait impressions from faces either wearing the mask or not, relevant limitations should nonetheless be stressed. Most notably, findings are based on a restricted number of face stimuli. Future studies will need to assess judgments about larger sets of faces, and this will also potentially allow to model the interaction between facial cues and contextual information. In addition, we employed static images of faces that may not fully capture the complexity and dynamic nature of social interactions. An endeavor for future research would be to rely on videos or, possibly, on face-to-face interactions. This would allow not only to manipulate the background scenarios in a more ecologically valid way, but also to introduce the manipulation of novel factors, such as the specific behaviors performed by the actor. For instance, first impressions from faces differ as a function of whether the target is stationary or is moving toward the viewer (Trifonova et al., 2024), and it would thus be important to outline how these dynamic factors further intervene in modulating the perception of mask wearers.

A further limitation the present work is that we only relied on explicit reports about trait impressions from faces. There is some evidence that the presence (vs absence) of face masks may affect deliberate behavioral intentions but not necessarily more automatic responses (Diekhof et al., 2024), thus raising the question about whether the contextual modulations observed in the present studies can also be detected through tasks that assess less controlled responses (e.g., priming tasks; Wentura & Degner, 2010).

To conclude, in both studies faces with a mask triggered more positive trait impressions as compared to faces without the masks. Importantly, however, this finding was modulated by contextual factors, pointing to the importance of considering trait impressions from faces as context-dependent and highly flexible (see Hehman et al., 2019). Hence, our studies contribute to scientific literature by providing evidence that any specific finding should be contextualized in terms of time and space, and results help shedding light on the complexity of trait impressions from faces during a global pandemic.

## Data Availability

The datasets generated during the current study are available in the OSF repository, https://osf.io/xtgnj/?view_only=519fffa90f2745e9bc639c5867593afa.

## Footnotes

1 Importantly, wearing face masks was mandatory in Italy in all indoor public spaces (e.g., shops, schools, buses) until May 2022. Afterward, such strict requirements were removed, and obligations remained active only in hospitals and other high-risk settings.

2 We first compared the trait impressions from faces reported at T1 by participants who took part in both phases of the Study (N = 169) with those of participants who responded only at T1 (N = 31). We ran a 2 (Face mask: present vs. absent) × 5 (Trait: trustworthiness, morality, sociability, competence, and altruism) × 2 (Involvement: only at T1 vs. at both T1 and T2) ANOVA with the latter factor between participants. Results showed that the Involvement factor did not give rise to any significant effect (all *p*s > 0.215).

3 Data analysis was conducted both with SPSS (v. 28) and with Jamovi (v. 2.3.28). Since our data were not normally distributed, we also carried out non-parametric tests to check for the robustness of our findings. In both Study 1 and Study 2, results were consistent with those emerging from the ANOVAs. For both Study 1 and 2 we used the Bonferroni correction from SPSS (v. 28) for the main comparison between factors.

4 Participants were subsequently also presented with other questions related to their appraisal of the pandemic that are not relevant with respect to the goals of the present study. However, the questions are presented in the Supplementary Material.
